# Automated Pain Spots Recognition Algorithm Provided by a Web Service–Based Platform: Instrument Validation Study

**DOI:** 10.2196/53119

**Published:** 2024-08-27

**Authors:** Corrado Cescon, Giuseppe Landolfi, Niko Bonomi, Marco Derboni, Vincenzo Giuffrida, Andrea Emilio Rizzoli, Paolo Maino, Eva Koetsier, Marco Barbero

**Affiliations:** 1 Rehabilitation Research Laboratory 2rLab, Department of Business Economics, Health and Social Care, University of Applied Sciences and Arts of Southern Switzerland, Via Violino 11, Manno, 6928, Switzerland, 41 586666442; 2 Institute of Systems and Technologies for Sustainable Production, University of Applied Sciences and Arts of Southern Switzerland, Lugano, Switzerland; 3 IDSIA Dalle Molle Institute for Artificial Intelligence, USI-SUPSI, Lugano, Switzerland; 4 Pain Management Center, Division of Anaesthesiology, Department of Acute Medicine, Neurocenter of Southern Switzerland, Regional Hospital of Lugano, Lugano, Switzerland

**Keywords:** pain drawing, image processing, body charts, scan, pain, draw, drawing, scanner, scanners, app, apps, applications, device, devices, image, images, smartphone, smartphones, scale, musculoskeletal, body chart, accuracy, reliability, accurate, reliable, picture, pictures, mobile phone

## Abstract

**Background:**

Understanding the causes and mechanisms underlying musculoskeletal pain is crucial for developing effective treatments and improving patient outcomes. Self-report measures, such as the Pain Drawing Scale, involve individuals rating their level of pain on a scale. In this technique, individuals color the area where they experience pain, and the resulting picture is rated based on the depicted pain intensity. Analyzing pain drawings (PDs) typically involves measuring the size of the pain region. There are several studies focusing on assessing the clinical use of PDs, and now, with the introduction of digital PDs, the usability and reliability of these platforms need validation. Comparative studies between traditional and digital PDs have shown good agreement and reliability. The evolution of PD acquisition over the last 2 decades mirrors the commercialization of digital technologies. However, the pen-on-paper approach seems to be more accepted by patients, but there is currently no standardized method for scanning PDs.

**Objective:**

The objective of this study was to evaluate the accuracy of PD analysis performed by a web platform using various digital scanners. The primary goal was to demonstrate that simple and affordable mobile devices can be used to acquire PDs without losing important information.

**Methods:**

Two sets of PDs were generated: one with the addition of 216 colored circles and another composed of various red shapes distributed randomly on a frontal view body chart of an adult male. These drawings were then printed in color on A4 sheets, including QR codes at the corners in order to allow automatic alignment, and subsequently scanned using different devices and apps. The scanners used were flatbed scanners of different sizes and prices (professional, portable flatbed, and home printer or scanner), smartphones with varying price ranges, and 6 virtual scanner apps. The acquisitions were made under normal light conditions by the same operator.

**Results:**

High-saturation colors, such as red, cyan, magenta, and yellow, were accurately identified by all devices. The percentage error for small, medium, and large pain spots was consistently below 20% for all devices, with smaller values associated with larger areas. In addition, a significant negative correlation was observed between the percentage of error and spot size (*R*=−0.237; *P*=.04). The proposed platform proved to be robust and reliable for acquiring paper PDs via a wide range of scanning devices.

**Conclusions:**

This study demonstrates that a web platform can accurately analyze PDs acquired through various digital scanners. The findings support the use of simple and cost-effective mobile devices for PD acquisition without compromising the quality of data. Standardizing the scanning process using the proposed platform can contribute to more efficient and consistent PD analysis in clinical and research settings.

## Introduction

Musculoskeletal pain is a frequent problem that affects a significant portion of the population and can have a major impact on quality of life [1]. Understanding the causes and mechanisms underlying musculoskeletal pain is crucial for the development of effective treatments and enhancement of patient outcomes. Moreover, investigating musculoskeletal pain contributes to the advancement of our understanding of anatomy, physiology, and pain mechanisms, with potential implications for comprehending and managing pain [2,3].

There are several methods for measuring muscle pain, including self-report measures, behavioral measures, and physiological measures [4]. Self-report measures involve asking the person to rate his or her level of pain on a scale, such as the Visual Analog Scale or the Numeric Rating Scale [5]. A promising way of evaluating pain using drawings is known as the Pain Drawing Scale [6-10].

Digital technologies have had a significant influence on the evolution of pain drawings (PDs), with different applications in the field of medical apps [11].

The fields of application of PDs include diagnosis of low back pain disorders; paresthesias evoked by implanted neurological stimulators; depiction of orofacial pain, such as headaches and toothaches; and evaluation of users of electric wheelchairs with pain located in the back, buttocks region, and so forth.

Body charts can also illustrate other types of sensory experiences such as numbness, tingling, hypoesthesia, or allodynia [12].

While digitally acquired PDs offer advantages, many studies demonstrate sophisticated analyses of scanned or digitized pen-and-paper PDs, showcasing the versatility of digital image processing. This capability enables the digitization and analysis of extensive collections of pen-and-paper pain diagrams, making it adaptable to various settings and needs.

In this technique, the individuals are instructed to color the area where they are experiencing pain, and the picture is then rated on a scale based on the amount of pain depicted [13]. This can be a useful tool for measuring pain in individuals who have difficulty verbalizing their pain experience, such as young children or nonverbal individuals [14]. However, it is important to keep in mind that PDs can be subjective and may be influenced by factors such as the person’s cultural background or level of education [15]. PDs typically consist of body charts with different views of the human body (dorsal, ventral, and side) or subportions (head, hand, etc), and patients are instructed to color with a marker the area where they experience pain. The body charts can be divided into regions such as the Margolis regions [16]. This technique is used to describe and categorize the location of musculoskeletal pain in the body. The regions are based on anatomical divisions of the body, including the neck, upper extremities, low back, and lower extremities [17].

The purpose of the Margolis regions is to provide a standardized and easily understood way to describe the location of musculoskeletal pain, which can help with diagnosis and treatment planning [18,19].

PDs can be analyzed in a variety of ways, depending on the purpose of the analysis and the method used to create the drawings. The most common method of analyzing PDs is the measurement of the size of the pain region. This can be done using computer software or manual measurement techniques [20].

There are different software programs available for analyzing PDs [21-25]. These programs can be used to perform both qualitative and quantitative analyses of PDs, depending on the specific software and the features it offers [26]. Some researchers also introduced sex-specific body charts in order to facilitate the communication of pain for women [17,27,28].

Some of the features offered by pain analysis software may include image digitization (allowing the conversion of traditional paper drawings into digital format for analysis), image scaling (allowing the adjustment of the size of the PDs to match a reference scale), image analysis (using algorithms to automatically identify and quantify features of the PD, such as the size and shape of the pain region), and data visualization (displaying the results of the analysis in a clear and easy-to-understand format, such as graphs or heat maps).

Submitting paper PDs to patients is simpler than using drawing applications running on tablets. Anyway, the use of PDs is not indicated in patients with vision impairment or in preschool children, although some studies investigated the application in teenagers. Being a self-assessed measure the patients should not have cognitive impairments, or diseases including misperception of their body.

While digital drawings can be easily edited and manipulated, and the tools available on a tablet can offer a wider range of color options and effects, paper PDs are largely used in clinical settings. This preference stems from the fact that many patients feel more comfortable using the pen-on-paper approach rather than digital devices [29-31].

There are different methods for scanning PDs, including using a flatbed scanner, a device that scans flat, thin documents placed on a glass window; a handheld scanner, a portable device that can scan images while being moved over them; a drum scanner, a high-end scanner that uses a rotating cylinder to capture the image; a multifunctional printer scanner, a printer that also includes a scanner function; and a virtual scanner, a software that can use a camera to scan images.

To date, there is no standardization in scanning PDs. The existing softwares for PD acquisition work with specific body charts and do not allow a direct comparison between using the same drawing. The aim of this study was to evaluate the accuracy of PD analysis performed by a web platform using different digital scanners. The objective of this study was to demonstrate that simple and relatively cheap mobile devices can be used to acquire PDs without loss of information.

## Methods

### Ethical Considerations

We did not involve patients, subjects, or animals. The data set was generated through a computer simulation; thus, there was no need to have ethical approval.

### Sketch Your Pain Platform

The proposed analysis was performed using a web platform. The main features of this distributed web application (currently available on a local server [32]) are as follows:

1. *Knowledge-base management*: the platform allows the collection of patient’s data (biometric, pain history, applied therapies, diagnoses, etc).
2. *PD acquisition*: PDs can be uploaded both digitally and from paper (see details in the following section).
3. *Basic PD analysis*: each pain spot is analyzed individually (ie, number of pixels, barycenter, etc).
4. *Smart analysis services*: the platform provides a plug-in–based mechanism that allows the implementation of additional analysis within the platform. In this way, researchers can apply specific innovative tools to the PDs stored in the database [33].

A paper PD can be imported in two ways: (1) it can be digitally imported by using the specific acquisition tool that allows for drawing directly on a tablet, using a digital pen on the touch screen (available on a local server [34]), or (2) it can be manually imported as a PDF file (the platform allows one to download a PDF file including empty body charts with a unique QR code, which can be printed, filled manually using a color marker, and scanned as a PDF file).

When a PD is generated digitally, it is already aligned with the body charts, while for the paper drawings, the process is more complex and can be summarized as follows. The body chart and all related information are identified thanks to the QR code, including the information of the protocol, subject ID, gender, and view of the body chart, and are stored in a database. The platform code and data are stored on a local server. The patient names and sensitive data are anonymized using codes that are available only to the operators.

**Figure 1.**
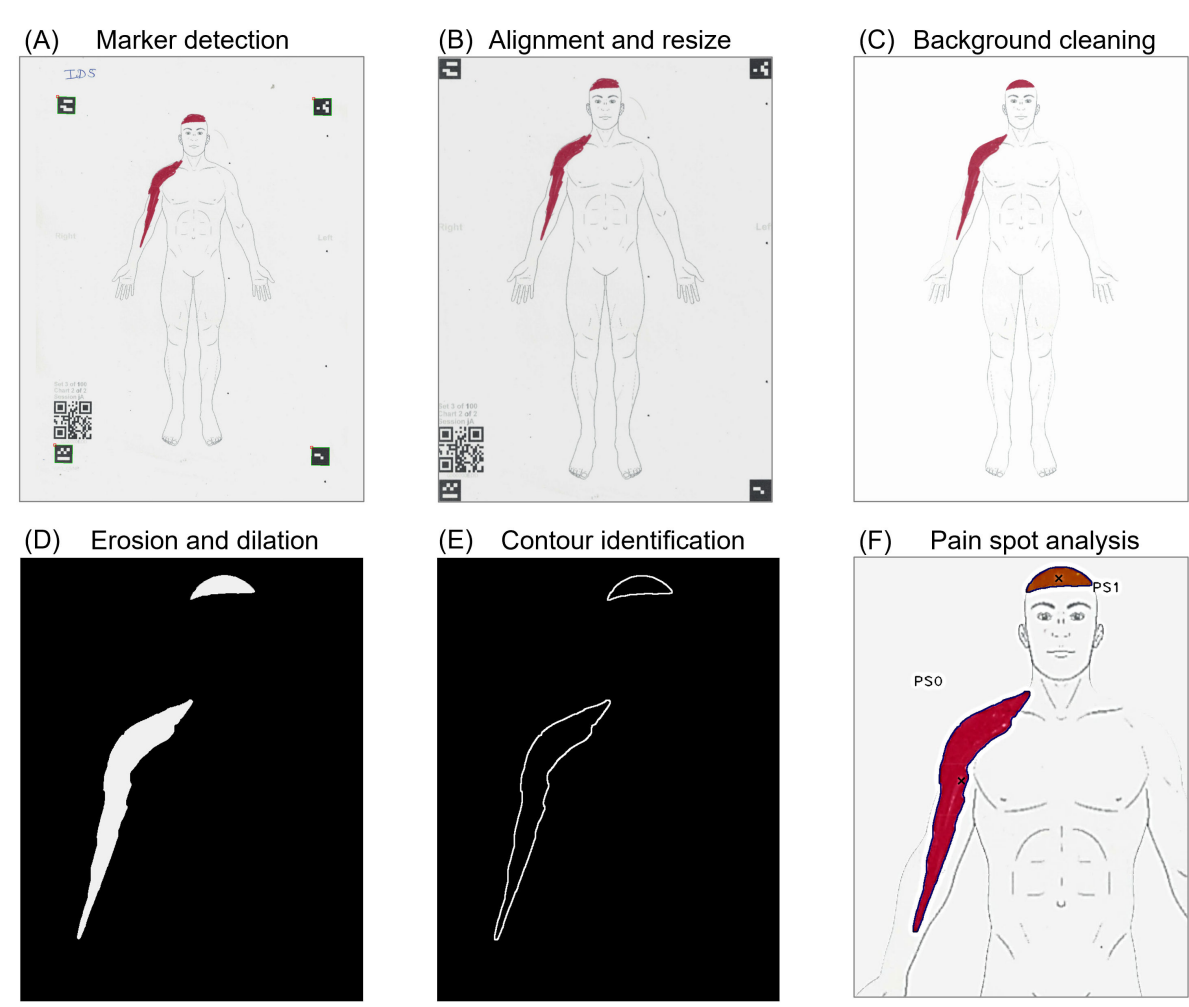
Pain spot detection process. (A) The 4 markers at the corners and the QR code are identified. (B) The pain drawing is aligned and scaled. (C) The areas outside the body chart are removed. (D) Pain drawings are separated from the background and eroded in order to correct the imperfections due to pen drawing. (E) Pain spot contours are identified and small holes are removed. (F) Each pain spot is analyzed to extract area and position. PS: pain spot.

The scanned image is aligned and cropped using the 4 markers at the corners as pivots (Figure 1A).

The image is resized in order to have the same number of pixels of the body charts stored in the platform (ie, 2048×1536 pixels; Figure 1B). The areas outside the body chart are removed using a mask image. This step also allows for the removal of all possible out-of-body staining errors (Figure 1C). The pain spots (that should be drawn in color and not in any shade of gray) are identified and isolated from the body chart by computing the SD of each pixel (in this way, the SD of black pixels [0, 0, 0] and white pixels [255, 255, 255] is equal to 0, while a red pixel [255, 0, 0] has an SD of 147.2). The optimal threshold for the minimum SD (based on preliminary tests) that works best in extracting pain spots from the body chart with different conditions of light and colors is 25. In this way, the color image is converted into a Boolean matrix where ones correspond to pixels with pain. An algorithm for extrusion and subsequent erosion is applied to the Boolean image in order to fill possible gaps that can happen when the user is using a sharp marker (Figure 1D). All contours of the pain spots and the potential holes in them are identified by means of the Canny edge detection algorithm [35]. The pain spots whose contours contain fewer than 20 points/pixels are removed. Likewise, the holes present in the spots, whose contours contain less than 15 points/pixels, are removed. Further, the pain spots smaller than 100 pixels are removed. Likewise, the holes smaller than 150 pixels are removed (Figure 1E). The individual pain spots are identified by an image segmentation algorithm, and for each spot, the area in pixels and the coordinates of the centroid are computed. The final result of the process is shown in Figure 1F.

### Generation of Artificial PDs

For the present protocol, 2 sets of PDs were generated with a homemade MATLAB (MathWorks) code. We decided to test the platform by using artificial PDs in order to have complete control of the process and of the analysis. For each of the pain spots generated randomly, we had information on pain location (barycenter of the pain spot), area in square pixels, and shade of color in the red, green, and blue (RGB) scale, and with these data, we could assess the performance of each of the scanning devices. A preliminary study was conducted on the platform using different scanning devices on PDs of real patients with similar results [36].

The body chart selected was a male frontal body chart, representing the contours of a full male body in frontal view (dimensions: 1536×2048 pixels).

### Color Analysis

**Figure 2.**
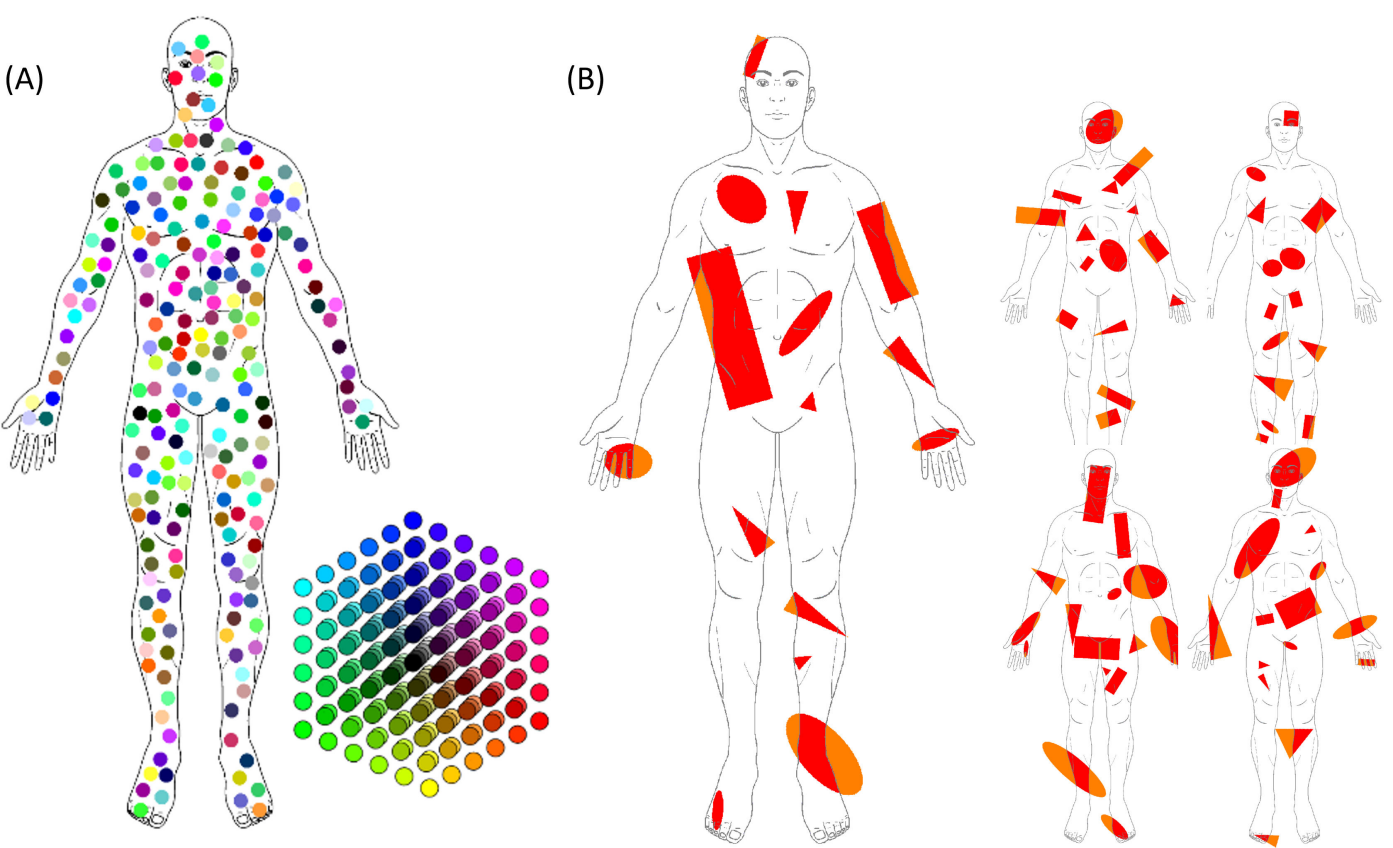
Representation of the artificial pain drawings generated with MATLAB. (A) A total of 216 colored circles with a 30-pixel diameter were randomly located within the area of the body chart. The colors were uniformly distributed in the RGB color cube. (B) Five body charts with randomly generated shapes. RGB: red, green, and blue.

The first artificial PD was generated, adding 216 colored circles, which were 33 pixels in diameter, within the body chart map. The circles were randomly positioned within the body chart in order to be nonoverlapping and not touching each other. The colors were chosen in order to uniformly span the RGB color cube, using 6 different intensities for each color. Since the color depth was defined on a range from 0 to 255 (1 byte), the values of each color were 0, 51, 102, 153, 204, and 255. In this way, the total number of colors was 6^3=216, ranging from black (0, 0, 0) to white (255, 255, 255) and including all combinations of RGB (ie, [0, 0, 0], [0, 0, 51], [0, 0, 102],…[51, 0, 0],…[255, 255, 255]; Figure 2A).

### Area and Location Analysis

The second set of PDs was composed of 5 artificial PDs generated by adding several red shapes (ellipses, rectangles, and triangles) to the same body chart; the shapes were generated with random sizes, orientations, and positions and could overlap and be partially outside the body chart mask (Figure 2B). The red color was chosen mainly because it can be easily associated with pain in a body chart; in addition, in the RGB cube, the red color is located in one of the vertices (ie, it has the highest SD value among triplets of RGB values, together with yellow, magenta, cyan, blue, and green), and it is easy to find red pens or markers in common shops.

Each of the 2 sets was printed in color, using the same printer (Sharp MX-7580) to print 11 copies; markers were added at the 4 corners, and a QR code was added at the bottom left side. The markers and the QR code were included in order to allow the platform to align the images and add the PDs to the internal database.

### Selection of Scanning Devices

The 11 sets of drawings were then scanned using different devices and apps (Table 1).

We selected 3 flatbed scanners with different sizes and prices: 1 professional office printer or scanner that was available in our university (Sharp MX-4070; price about US $5000), 1 portable flatbed scanner (Canon Lide 220; ~US $50), and a home printer or scanner (HP Envy 4500; ~US $300).

In addition, we selected 3 smartphones with different price ranges: iPhone 12 (~US $1000), Samsung Galaxy (~US $400), and Ulefone Armor (~US $100). All the 3 devices were using the same app for scanning images (vFlat scan), in order to compare only the hardware of the devices.

Moreover, for the cheapest smartphone, we selected 6 free apps available in the android apps Google Play repository. The apps were selected according to their popularity and ranking based on users’ comments.

For each scanner, a PDF file was generated including the corresponding set of images (1 with colored circles and 5 with red shapes). The PDF files were uploaded in the sketch your pain platform [32].

**Table 1.** List of devices used to scan the artificial pain drawings^a^.

Type	Device model	Resolution	Price (US $)	App
Flatbed	Sharp MX-4070	300 dpi^b^	~5000	—^c^
Flatbed	Canon Lide 220	300 dpi	~50	—
Flatbed	HP Envy 4500	300 dpi	~300	—
Smartphone	iPhone 12	12 Mpx^d^	~1000	vFlat Scan
Smartphone	Samsung Galaxy S10 Lite	32 Mpx	~400	vFlat Scan
Smartphone	Ulefone Armor-X	13 Mpx	~100	vFlat Scan
Smartphone	Ulefone Armor-X	13 Mpx	~100	TapScanner
Smartphone	Ulefone Armor-X	13 Mpx	~100	Simple Scan
Smartphone	Ulefone Armor-X	13 Mpx	~100	Fast Scanner
Smartphone	Ulefone Armor-X	13 Mpx	~100	CamScanner
Smartphone	Ulefone Armor-X	13 Mpx	~100	TurboScan

a The flatbed devices generated the PDF files using proprietary software, while smartphones needed an app to generate the PDF using the camera.

b dpi: dots per inch.

c Not available.

d Mpx: megapixels.

### Image Processing

The original area of each pain spot generated with MATLAB was computed as well as the coordinates of the centroid of each pain spot. The sketch your pain platform identified the QR codes and allowed the processing of each identified pain spot, providing area in pixels and coordinates of the centroid.

**Figure 3.**
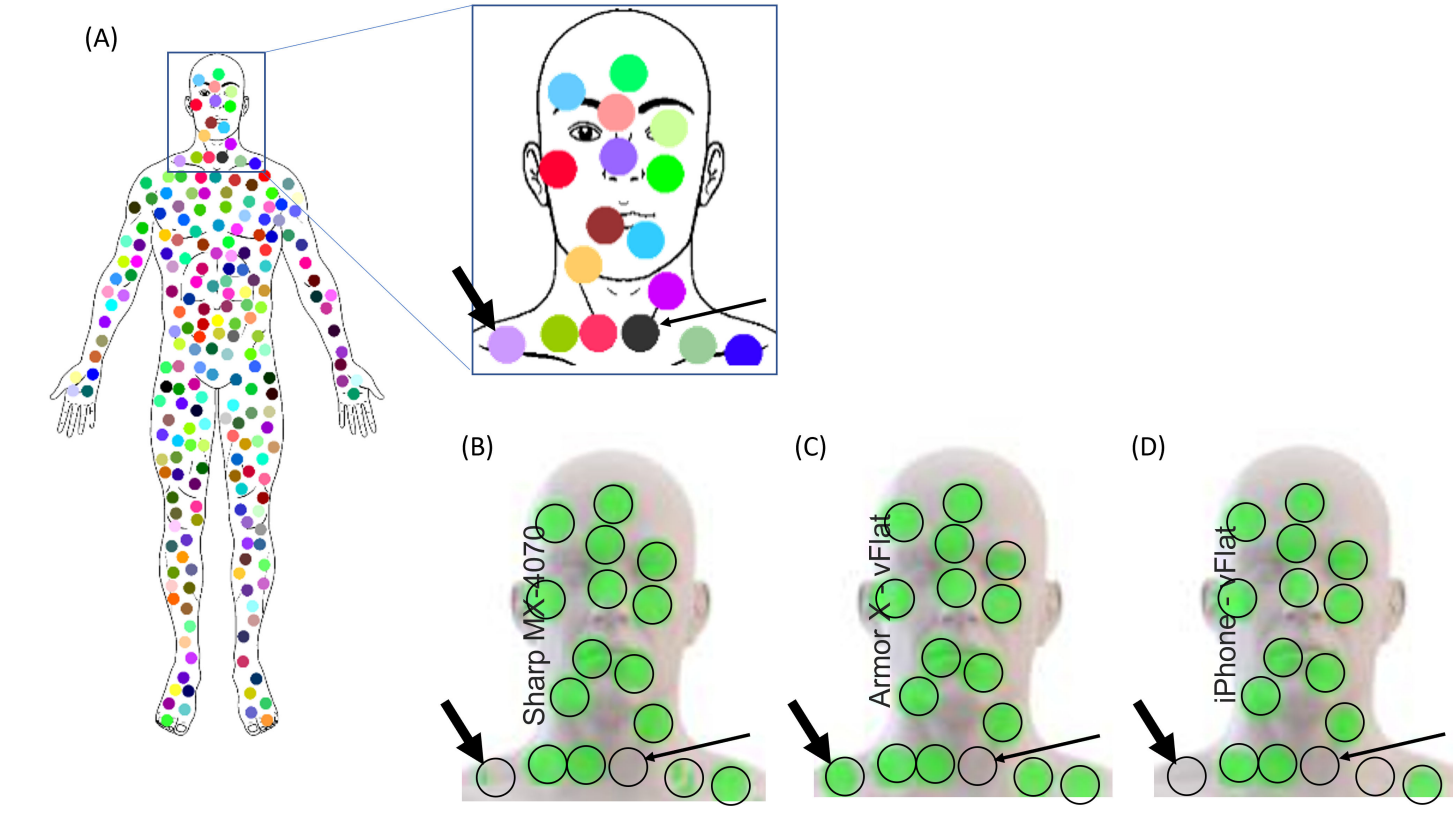
Examples of identification of pain spots from colored circles. (A) The original drawing and the output of the platform algorithm for 3 different devices are shown. The green color represents an identified pain spot. The purple circle on the bottom left corner (indicated with the thick arrow) was not identified by the iPhone (D) and Sharp scanner (B), but it was identified by the Armor phone (C). The black circle on the neck (indicated with the thin arrow) was not identified by any of the devices.

For the analysis of colors, we analyzed whether the platform was able to identify a pain spot corresponding to each of the locations where colored circles were generated. If the area of the identified pain spot was larger than a fixed threshold (90% of the theoretical area; eg, 450 out of 500 pixels), then the pain spot was counted (Figure 3).

### Statistical Analysis

The variables used for the statistics were the area (A) of pain spots in square pixels and the coordinates of the centroid (x, y) of each pain spot in pixels. The variables computed for each scanning device were compared with the variables computed for the corresponding pain spots on the original artificial PDs generated with MATLAB. The percentage area error (E) was computed as the difference between the 2 areas divided by the area computed on original PD and expressed as a percentage. The distance (D) between the theoretical centroid of the pain spot computed on original image and the centroid of the pain spot identified by each device was also computed and expressed in pixels.

Intraclass correlation coefficient (ICC) estimates (and their 95% CIs) of pain area and barycenter coordinates were calculated using MATLAB and a 1-way mixed-effects model.

In addition, standard error of measurement (SEM) and minimal clinical differences were computed for pain area and barycenter position.

Descriptive statistics is presented with box and whisker plots with median and IQR values.

## Results

### Color Analysis

**Figure 4.**
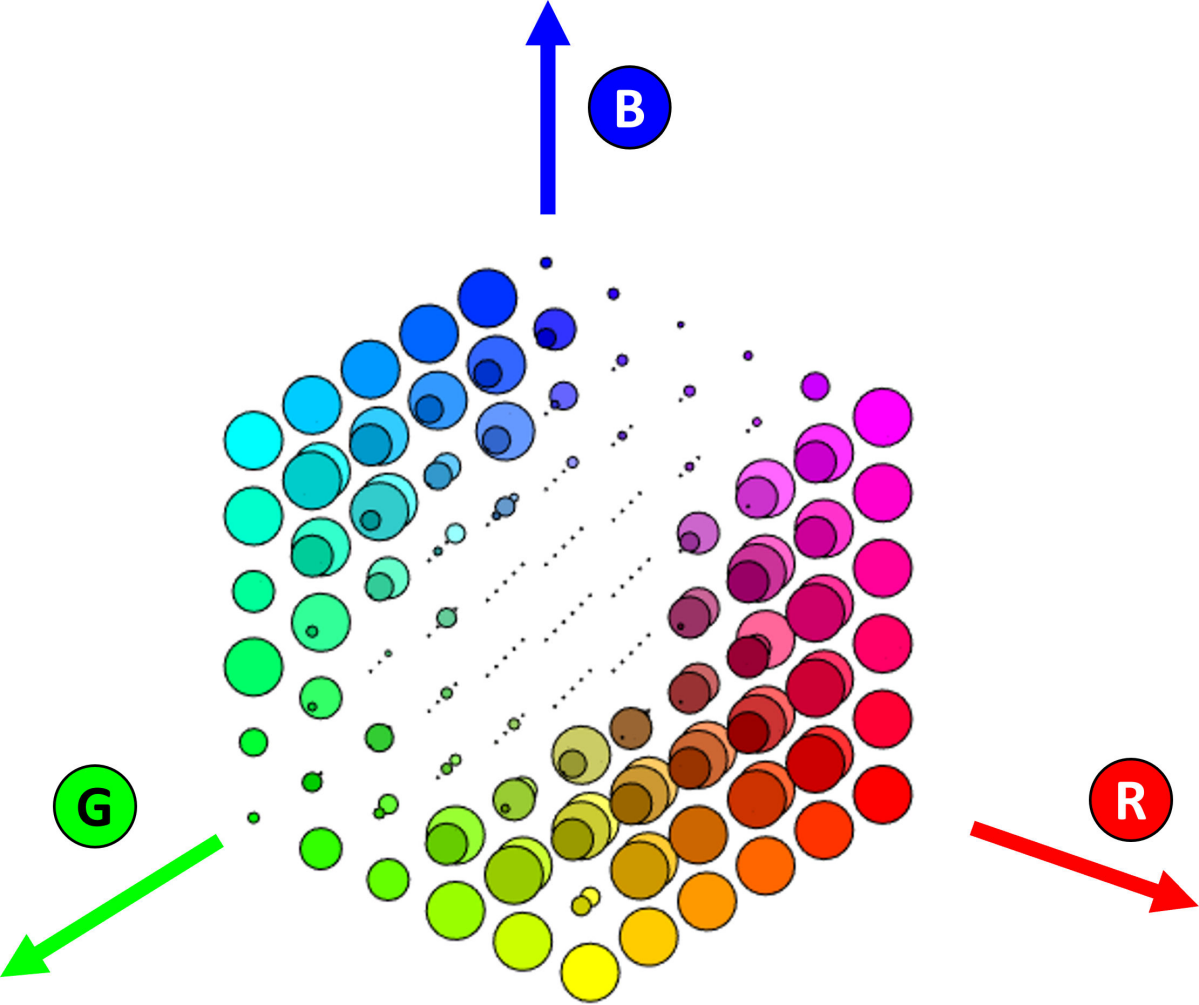
Representation of the performance of the algorithm in identifying different color circles. The middle diagonal (from white to black where RGB components are equal) and the colors located close to the central diagonal are not identified by the algorithm, while colors such as red, magenta, and yellow are identified by all devices. RGB: red, green, and blue.

A Boolean table with the results of pain spot identification was generated with 216 lines (1 for each color) and 11 columns (1 for each scanner). A graphical representation of the table is represented in Figure 4.

Each color is represented by a circle in the 3D color cube, and the size of each circle is proportional to the number of scanners that were able to identify that color (ie, if the area of the identified pain spot was larger than 90% of the printed circle). The maximum circle diameter was set to two-thirds of the spacing between adjacent circles. In this way, it is easy see which colors are best for PDs. As expected, the colors with identical values in RGB triplets (ie, shades of gray, black, and white) were never identified by the software, and colors with low values of SD in RGB triplets (eg, low-saturation colors) were not identified with most devices. The colors that were identified with all devices are located close to the corners of the color cube (ie, high-saturation colors, such as red, cyan, magenta, and yellow).

### Area and Location Analysis

**Figure 5.**
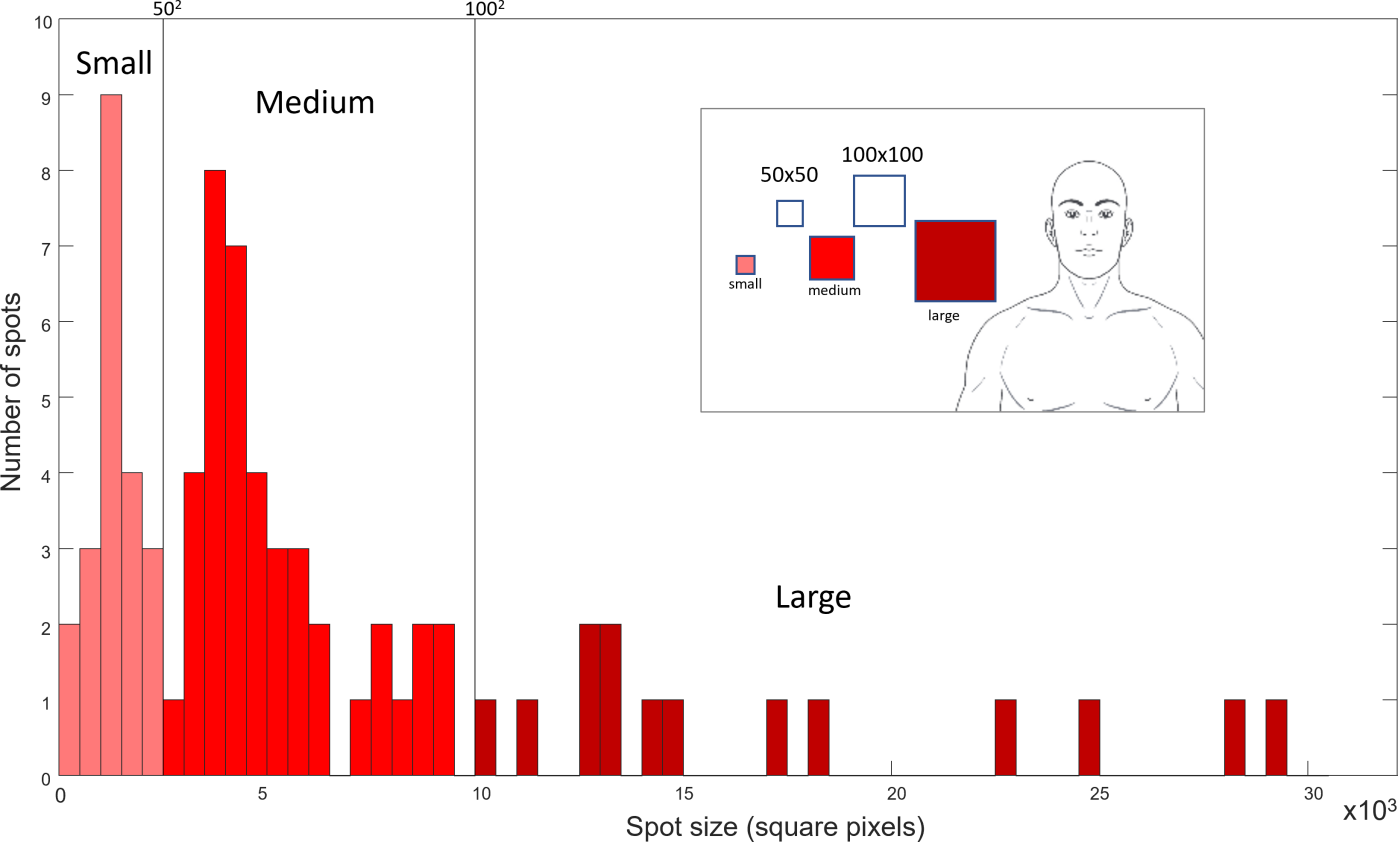
Representation of the distribution of shapes according to their size. The 3 colors are used to represent the 3 categories that were used for further analysis The image in the legend shows the thresholds used to divide the categories (as square shapes).

Figure 5 shows the distribution of areas of the pain spots generated artificially and randomly distributed on each of the 5 body charts. The pain spots were divided into 3 categories according to their area in square pixels (A<50^2^: small; 50^2^≤A<100^2^: medium; and A≥100^2^: large).

The ICC for pain area was 0.99, with a 95% CI 0.99‐0.99 (*F*_74,750_=1.44e+04). In addition, the ICC and CI values for barycenter coordinates were above 0.99 (x-coordinate: *F*_84,930_=4.55e+05; y-coordinate: *F*_103,843_=5.12e+05).

Table 2 shows the SEM and minimal detectable change values for each device compared with the theoretical value.

**Figure 6.**
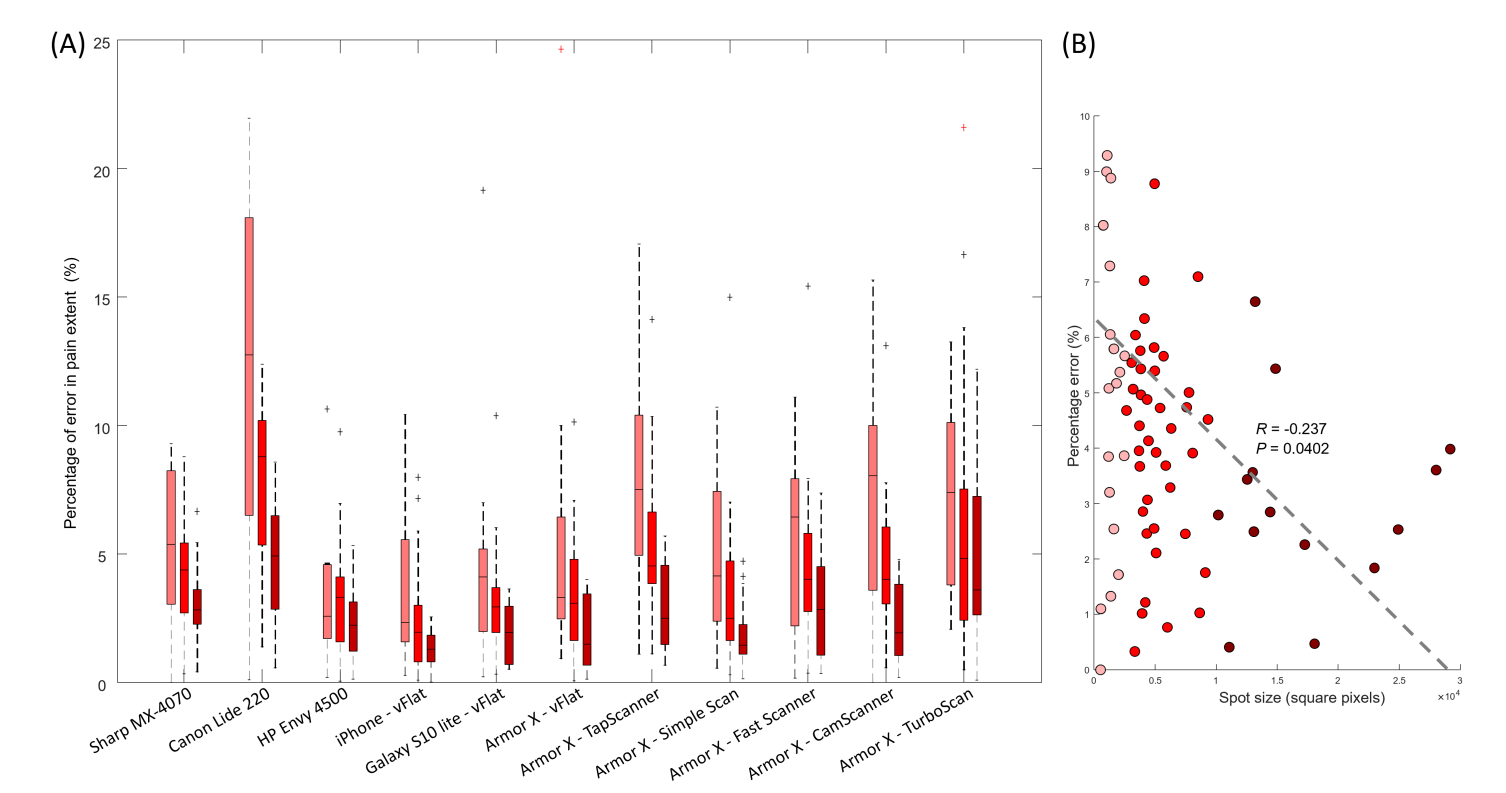
(A) Distribution of errors in identifying the pain spot areas expressed in percentages. The 3-color box and whisker plots for each device represent the distribution of area error for each of the 3 categories (small, medium, and large pain spots). (B) Correlation between percentage of error and spot size (the regression line is indicated as a dashed line).

Figure 6A shows the percentage error of pain extent for each device for the 3 categories of pain spot areas. For all devices, the percentage error was below 20% for small, medium, and big pain spots, with lower values associated with bigger areas. A significant negative correlation was observed between percentage of error and spot size (*R*=−0.237; *P*=.04; Figure 6B).

**Figure 7.**
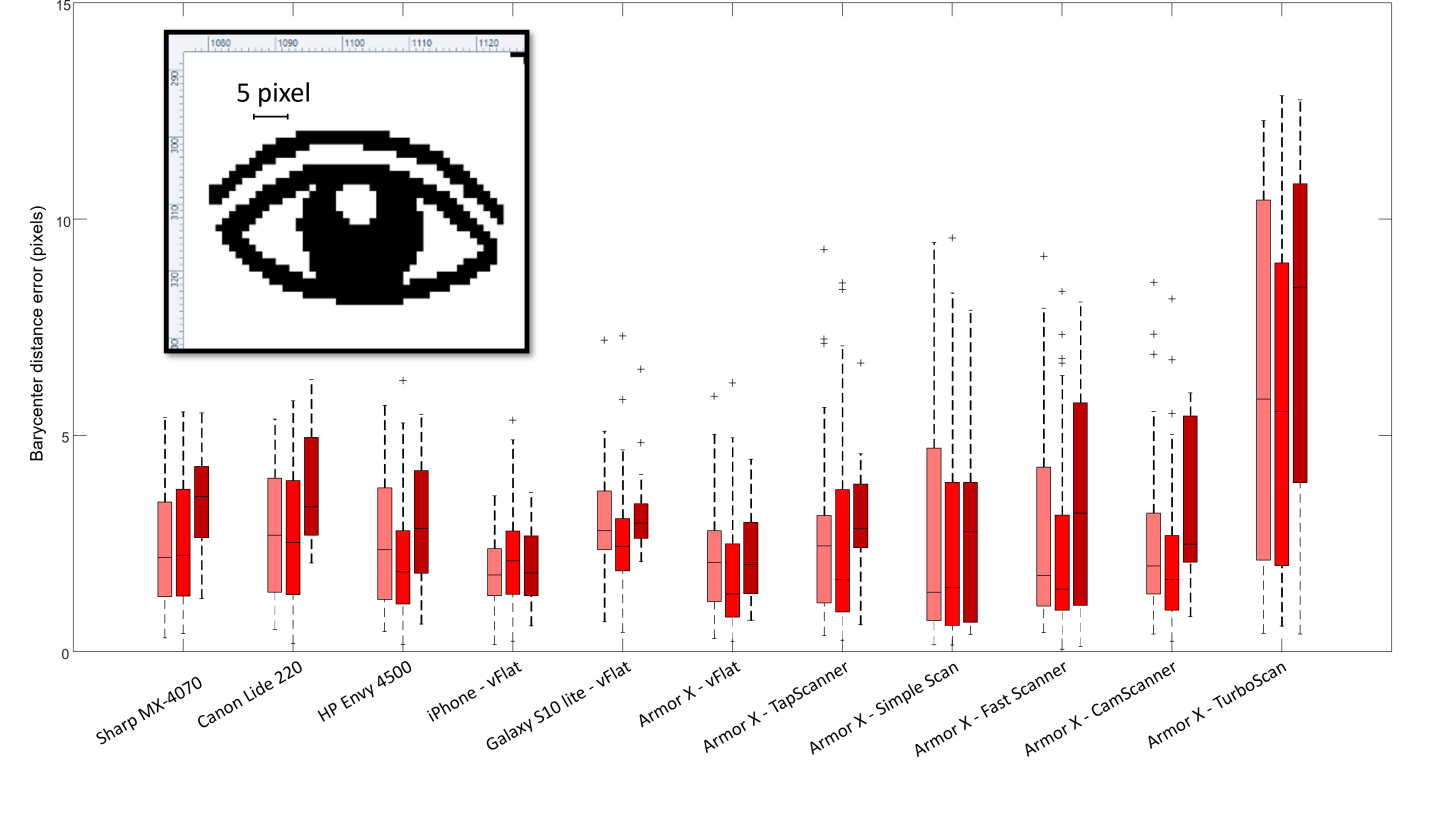
Distribution of errors in the location of the pain spots expressed in pixels. The 3-color box and whisker plots for each device represent the distribution of barycenter distance error for each of the 3 categories (small, medium, and large pain spots). The image of the eye shows the actual dimensions of 5 pixels in the full body chart (paper dimensions: 2048×1536 pixels).

Figure 7 shows the percentage error of distance between the theoretical location of the centroid of each pain spot and the location of the centroid of the identified pain spot. The distribution of the distances was always below 5 pixels except for the Armor device with the TurboScan app (11th column).

**Table 2.** Standard error of measurement and minimal detectable change for the identification of pain area and for the barycenter distance for each device compared with the theoretical value.

Device model	Area SEM^a^ (pixels^2^)	Area MDC^b^ (pixels^2^)	Barycenter distance SEM (pixels)	Barycenter distance MDC (pixels)
Sharp MX-4070	262	513	1.5	2.9
Canon Lide 220	348	682	1.5	3.0
HP Envy 4500	219	429	1.5	3.0
iPhone—vFlat	134	262	1.0	2.1
Galaxy S10 lite—vFlat	172	336	1.4	2.6
Armor X—vFlat	182	356	1.3	2.5
Armor X—TapScanner	247	485	2.1	4.2
Armor X—Simple Scan	251	491	2.6	5.1
Armor X—Fast Scanner	287	563	2.4	4.8
Armor X—CamScanner	227	444	2.0	3.8
Armor X—TurboScan	505	989	4.0	7.8

a SEM: standard error of measurement.

b MDC: minimal detectable change.

## Discussion

### Principal Findings

The results of this study showed that the pen-and-paper drawings can be imported and processed with negligible differences using different devices. To our knowledge, there were no other studies focusing on the acquisition of paper pen drawings using mobile devices. Before conducting this study, we were largely using our platform, asking patients to use the red marker because we suspected that blue markers would be mistaken as black. As expected, indeed the red color is the best choice for multiple reasons: the red color is associated with the inflammatory process; thus, it is easy for a patient to visualize their own pain as a red spot (eg, compared with green or blue). Moreover, in shops, markers labeled as “red” are very similar to the theoretical value of (255, 0, 0), while markers labeled as “green,” or “blue” can have different darker or lighter shades.

Surprisingly, in our results, blue and green were identified only with few devices (mostly flatbed scanners). One of the reasons could be the ink of the printer, whose color was slightly different from what was expected. The illumination of the room could also have an impact, since the light emitted by different bulbs or neon could carry different wavelengths in different proportions. The light sensors of cameras and scanners could have different sensitivities to different wavelengths, and probably the red-light sensors have higher sensitivities.

When observing the printed page, we noticed that the blue ink was slightly darker than what we observed on the PC monitor, but it was difficult to objectively evaluate which one was correct, as we did not have a gold standard for each color. In summary, the results confirmed our initial prediction: the red marker is the optimal choice for PDs. However, in cases where the patient does not have a red marker available, we recommend using a flatbed scanner to generate the PDF for import to the platform. This approach helps minimize the bias resulting from external factors such as lights or photo LED sensitivity.

Regarding the accuracy of the pain spot identification, all the devices showed similar performance when using red color. The cheapest flatbed scanner showed larger errors probably due to the distortion of the image. When observing the digital image, we noticed that the proportions were slightly distorted (maybe due to the calibration of the motor or due to friction of the transmission chain). For this reason, the alignment process of the algorithm could not perfectly align the 4 markers in the corner; thus, the pain spot location had larger errors and extensions.

As expected, the smaller pain spots showed larger percentage errors but no differences in the barycenter location error, because of the distortion of pain spots due to the erosion process. The choice of the app for the mobile device had a significant effect on the percentage errors. In particular, the app named V-Flat showed better results than the others (when installed on the cheapest mobile device), leading to results that were comparable with high-rank mobile devices. The V-Flat app includes an algorithm that recognizes the corners of the paper and compensates for the distortions of the camera and even the distortions due to bent paper. For this reason, the results were as good as flatbed professional scanners (<5% of error). In general, the errors in identification of pain extent (<5%) and pain location (<5 pixels) were much lower than the precision of a subject in drawing or identifying their own pain and thus were lower than the minimum clinical significance of PDs [17,37,38].

### Limitations

This study has some limitations that may introduce bias into the results. First, the acquisitions made with mobile devices were not conducted under controlled lighting conditions. Although all acquisitions took place during daylight hours without direct sunlight on the paper, variations in the time of day and weather conditions could have affected the colors identified by the devices. In addition, we did not calibrate the “white level” of the mobile phone camera. While some apps offered advanced camera settings for optimizing virtual scanners, we chose to use the default settings to maintain as close to a real-life environment as possible. As a result, we did not test under unusual lighting conditions (eg, candlelight, colored lamps, neon lights).

Another limitation is that we could not directly compare the performance of our platform with other existing methods, since the body charts are specific for each existing app.

Furthermore, we tested only 1 printer to print all the artificial PDs, which introduces potential bias. The colored circles in the first part of the study were positioned within the body chart but in different locations. This variation in placement could impact the results, as colors farther from the center of the paper may experience greater distortion due to misalignment. However, this approach was necessary to avoid printing an excessive number of papers.

The decision of using artificial PDs is due to the fact that we conducted 2 studies in parallel. The first study involved the use of actual PDs generated by humans [36], while in this study, we wanted to investigate different colors in order to investigate all the RGB space. In addition, the location of pain spots in PDs generated by humans depends on the pathology of patients, while in this case, we preferred to have a uniform distribution of pain spots with a priori known sizes and locations. Both studies showed that the platform has excellent results, but in this study, we were able to quantify the error since we knew the theoretical pain spot areas.

The sample size of mobile devices and flatbed scanners is small. However, the objective of the study was not to provide an exhaustive sample of devices but rather to demonstrate that even inexpensive devices are sufficient for accurately acquiring paper PDs.

Similarly, the sample of scan apps is also limited, and some of them are no longer available for free as of manuscript submission. The app market is continuously evolving, with new apps being released regularly. Our intention was to find a selection of free virtual scanners available in the Play Store, and again, our aim was to show that various apps perform similarly.

### Conclusions

The system was already tested in real clinical settings and was shown to be easy to implement, easy to use, and well accepted. The acquisition of paper PDs using the proposed platform has been demonstrated to be robust and reliable across a wide range of scanning devices. The accuracy of pain extent and location analysis consistently falls within the error measurement range of PDs. The use of the proposed algorithm will enable the use of PD analysis in various clinical settings.

## Abbreviations

ICC: intraclass correlation coefficient
PD: pain drawing
RGB: red, green, and blue
SEM: standard error of measurement
